# 1250. Development of Linezolid and Daptomycin Resistance in Vancomycin Resistant *Enterococcus faecium* (VRE) during prolonged treatment for Intraabdominal Abscess

**DOI:** 10.1093/ofid/ofab466.1442

**Published:** 2021-12-04

**Authors:** Omar Al-Heeti, Tejas Joshi, William Justin Moore, Samuel W Gatesy, Nathan B Pincus, Kelly E R Bachta

**Affiliations:** 1 Northwestern University, champaign, Illinois; 2 Northwestern Feinberg School of Medicine, Chicago, Illinois; 3 Northwestern Medicine, Chicago, IL

## Abstract

**Background:**

Vancomycin-resistant enterococci (VRE) are nosocomial pathogens with extensive intrinsic and acquired antimicrobial resistance (AMR) mechanisms. We report a case in which intraabdominal (IA) and blood cultures grew linezolid and daptomycin resistant VRE (DLVRE).

**Methods:**

We report a case of DLVRE bacteremia after prolonged treatment with linezolid and daptomycin.

**Results:**

The patient was a 65-year-old female with a history of multiple abdominal surgeries who presented for elective incisional hernia repair. Her post-operative course was complicated by the development of loculated IA abscesses. A drain was placed into the largest abscess, and aspiration cultures were polymicrobial containing vancomycin-resistant *E. faecium* (Isolate 1). The patient was treated meropenem, fluconazole and linezolid for 6 weeks. Clinical and radiographic improvement was achieved. However, 4 days after competing antibiotics she developed recurrent abdominal pain and a leukocytosis. Daptomycin was chosen out of concern for long-term linezolid toxicity and IA cultures demonstrated new linezolid resistance (Isolate 2, LVRE). After an additional three weeks of therapy, she developed a catheter-associated bloodstream infection (CLABSI). Blood cultures revealed daptomycin-resistant LVRE bacteremia (Isolate 3, DLVRE). She was started empirically on a combination of ceftaroline and daptomycin, her PICC line removed, and her blood cultures cleared. Her antibiotic course is presented in Figure 1 and resistance patterns of the VRE in Table 1.

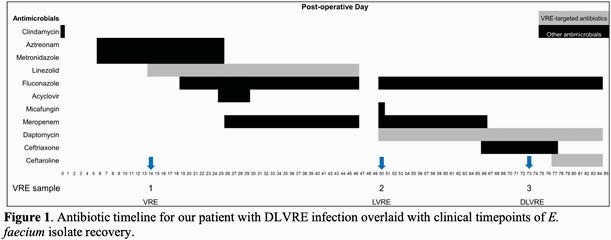

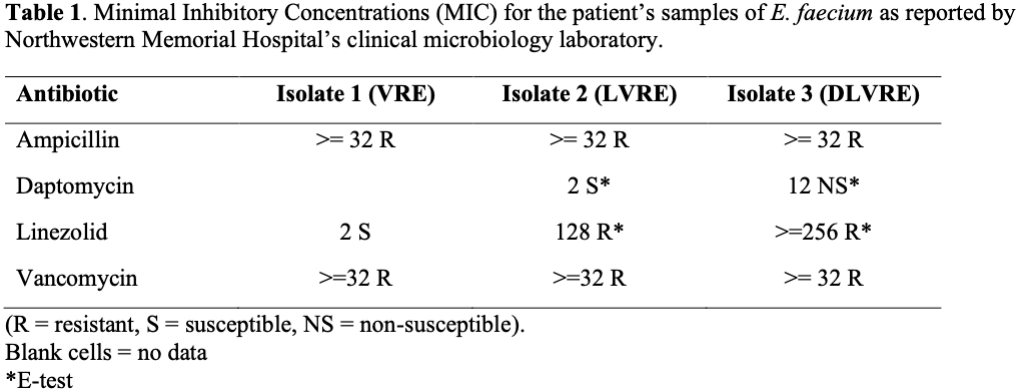

**Conclusion:**

In this patient, an IA abscess known to harbor VRE developed resistance to both linezolid and daptomycin during prolonged treatment with both agents. Ultimately, the patient experienced an episode of CLABSI DLVRE. Limited data exists on appropriate antibiotic choice in such challenging situations. Based on prior clinical and experimental data, we elected to use daptomycin in conjunction with ceftaroline for synergy, and the patient achieved the desired clinical response, clearance of her blood cultures and diminishing size of her IA abscess. Further work is needed to elucidate the best course of treatment for patients with VRE requiring long-term antibiotic therapy and for those who have developed extensively drug-resistant *E. faecium*.

**Disclosures:**

**All Authors**: No reported disclosures

